# 

**Published:** 1961-01

**Authors:** 


### Contents

| | | |
|---|---|---|
| DISEASES DUE TO MEDICAL TREATMENT | *C. Bruce Perry* | 1 |
| RESTORATIVE RESECTION OF THE RECTUM—ITS HISTORY AND PRESENT STATUS | *C. Patrick Sames* | 7 |
| THE GERIATRIC SERVICE | *T. W. Lloyd* | 12 |
| RESEARCH ON RHEUMATIC DISEASES | *G. D. Kersley* | 20 |
| CHRONIC OSTEOMYELITIS WITH MATURATION ARREST OF GRANULOCYTES | *Clinical Pathological Conference* | 25 |
| NOTES AND NEWS | | 35 |

